# Feedforward Inhibition Mediated by Preoptic Area Parvalbumin Neurons Contributes to Orexin-Induced Active Arousal from Anesthesia

**DOI:** 10.34133/research.1474

**Published:** 2026-11-05

**Authors:** Mingzi Ran, Rui Li, Haopeng Zhang, Huiming Li, Yuting He, Jing Zhen, Juan Guo, Guangchao Zhao, Mengmeng Li, Min Cai, Jiannan Li

**Affiliations:** ^1^ Department of Anesthesiology, The Fourth Medical Center, PLA General Hospital, Beijing, China.; ^2^ Department of Anesthesiology and Perioperative Medicine, Xijing Hospital, The Fourth Military Medical University, Xi’an, Shaanxi, China.; ^3^ Key Laboratory of Anesthesiology (The Fourth Military Medical University), Ministry of Education, Xi’an, Shaanxi, China.; ^4^ Department of Anesthesiology, School of Stomatology, The Fourth Military Medical University, Xi’an, Shaanxi, China.; ^5^ Department of Anesthesiology, Zhongshan Hospital, Fudan University, Shanghai, China.; ^6^ Department of Psychiatry, Xijing Hospital, The Fourth Military Medical University, Xi’an, Shaanxi, China.

## Abstract

The orexinergic system can actively promote arousal from general anesthesia, but the downstream circuit mechanisms that mediate this effect remain poorly defined. Orexin neurons densely innervate the sleep-promoting preoptic area (POA) of the hypothalamus, yet whether and how the POA contributes to orexin-induced arousal from general anesthesia is unknown. Using fiber photometry, optogenetics, neuropharmacology, and in vitro and in vivo electrophysiology in mice, we examined the orexin-to-POA circuit during isoflurane anesthesia and arousal process. We found that orexinergic inputs to the POA are suppressed by isoflurane anesthesia and recover before behavioral arousal. Enhancing POA orexinergic afferents markedly accelerated both behavioral and electrocorticographic arousal. This effect was mediated specifically by orexin type-1 receptors because local blockade of these receptors within the POA attenuated the arousal response. In situ hybridization and in vitro electrophysiology further implicated POA GABAergic neurons in this process. In vivo recordings revealed heterogeneous firing responses among POA GABAergic neurons, with excitation of a subset of parvalbumin-positive neurons preceding suppression of other GABAergic neurons, consistent with a feedforward inhibitory process contributing to orexin-induced arousal. These findings define a specific circuit mechanism for the transition from anesthesia to consciousness and suggest that parvalbumin neuron-associated feedforward inhibition within the POA mediates orexin-induced active arousal.

## Introduction

General anesthesia represents reversible loss of consciousness and alleviates pain in patients, thereby making essential contributions to modern clinical medicine [1]. Recovery of consciousness from general anesthesia is widely acknowledged not as a passive process following elimination of the anesthetics but as an active process regulated by complex molecular and neural circuit mechanisms [2,3]. This paradigm shift underscores the critical role of specific brain systems in the active rebooting of consciousness, with profound implications for addressing clinical complications such as delayed emergence and postoperative cognitive dysfunction [4,5].

Orexin neurons (also named hypocretin neurons), which mainly distribute in the lateral hypothalamus, play a pivotal role in stabilizing wakefulness [6,7] and triggering emergence of consciousness from general anesthesia [8]. Our previous studies indicated that orexin neurons can actively promote arousal from general anesthesia, as evidenced by increased expression of c-Fos (a well-established marker of neuronal activation) compared with the wakeful state prior to anesthesia [9]. This arousal-promoting effect of orexinergic neurons—which contributes to aging-related delayed emergence [10]—is mediated by their projections to and modulation of multiple nuclei within the ascending arousal system, including the ventral tegmental area (VTA), the nucleus accumbens, and the locus coeruleus [11].

Nevertheless, a key mechanistic gap remains. Most identified arousal-promoting systems are characterized by their modulatory effects on global arousal tone; yet, behavioral recovery of consciousness often manifests as a rapid, discrete transition rather than a slow, graded progression [3]. Notably, no single brain region appears to be solely responsible for emergence from anesthesia. How an arousal-promoting neuromodulatory system such as the orexin network converts apparently cumulative drive into a fast and stable switch between unconsciousness and consciousness is poorly understood. In particular, direct evidence is lacking on how neuronal firing timing and firing-pattern reorganization within local circuits underlie orexin-mediated active emergence. Interestingly, orexinergic neurons also send dense projections to the preoptic area (POA) [12], which consists mainly of GABAergic neurons that inhibit arousal centers thus promoting sleep [13]. Notably, real-time neuronal activity [14] and Fos expression [15] in the POA showed divergent changes during general anesthesia [16]. Thus, determining whether and how the POA neuronal firing dynamically mediates orexin-induced active emergence of consciousness from this deeply suppressed state remains one of the essential open questions.

To explore this fundamental knowledge gap and dissect the functional role of the orexin–POA circuit as a state-transition switch, we performed a multilevel investigation. First, we established physiological relevance by determining whether activity changes in POA orexinergic afferents correlate with anesthesia-induced alterations in consciousness states using in vivo fiber photometry, an optical approach for monitoring calcium-dependent fluorescence signals from genetically defined neural elements. Next, to address the causal question of whether modulating POA orexinergic afferent activity can directly control arousal, we used optogenetics, a light-based approach for activating or inhibiting genetically targeted neurons or axon terminals, to bidirectionally manipulate terminal activity, monitoring both behavioral and electrocorticography (ECoG) outcomes to validate this hypothesis. We further investigated the underlying mechanisms by identifying the key orexin receptor subtype through targeted pharmacology. Finally, to understand how these microcircuit-level effects are implemented at the cellular level, we used in vitro and in vivo electrophysiological recordings, directly characterizing, for the first time, the firing-pattern responses of population-level and subtype-specific POA GABAergic neurons to orexinergic activation. Our study reveals that orexin promotes emergence of consciousness from isoflurane general anesthesia by orchestrating a complex, diverse reorganization of the GABAergic microcircuitry within the POA, suggesting the orexin–POA pathway as a promising target for modulating consciousness states.

## Results

### Changes of POA orexinergic signal precede consciousness state transitions induced by isoflurane general anesthesia

To systematically investigate the functional role of the POA in orexin-induced active arousal from anesthesia, we first confirmed its anatomical and physiological relevance. We began by performing anterograde tracing (Fig. 1A). By injecting Cre-dependent virus with mCherry in the perifornical lateral hypothalamus (PeFLH) in Hcrt-Cre mice and orexin antibody immunofluorescence staining, we found that 90.93% [4.47%] of orexin-antibody-positive neurons express mCherry and 93.64% [3.43%] of mCherry-positive neurons express the orexin antibody (Fig. 1B). Whole-brain mapping results showed that orexin neurons sent dense projections to the POA, and the orexinergic axon density in coronal sections near bregma was 56.66% [4.23%] (Fig. 1C and D). Having established the anatomical connecting evidence, we next investigated the orexin receptor expression by performing in situ hybridization. We found that 86.67% [9.2%] of POA neurons express the orexin-1 receptor, 27.78% [8.7%] express the orexin-2 receptor, and 18.89% [6.3%] express both orexin-1/2 receptors (Figs. 1E to 3G). These results confirm the anatomical basis of the orexin neurons to the POA pathway.

**Fig. 1.**
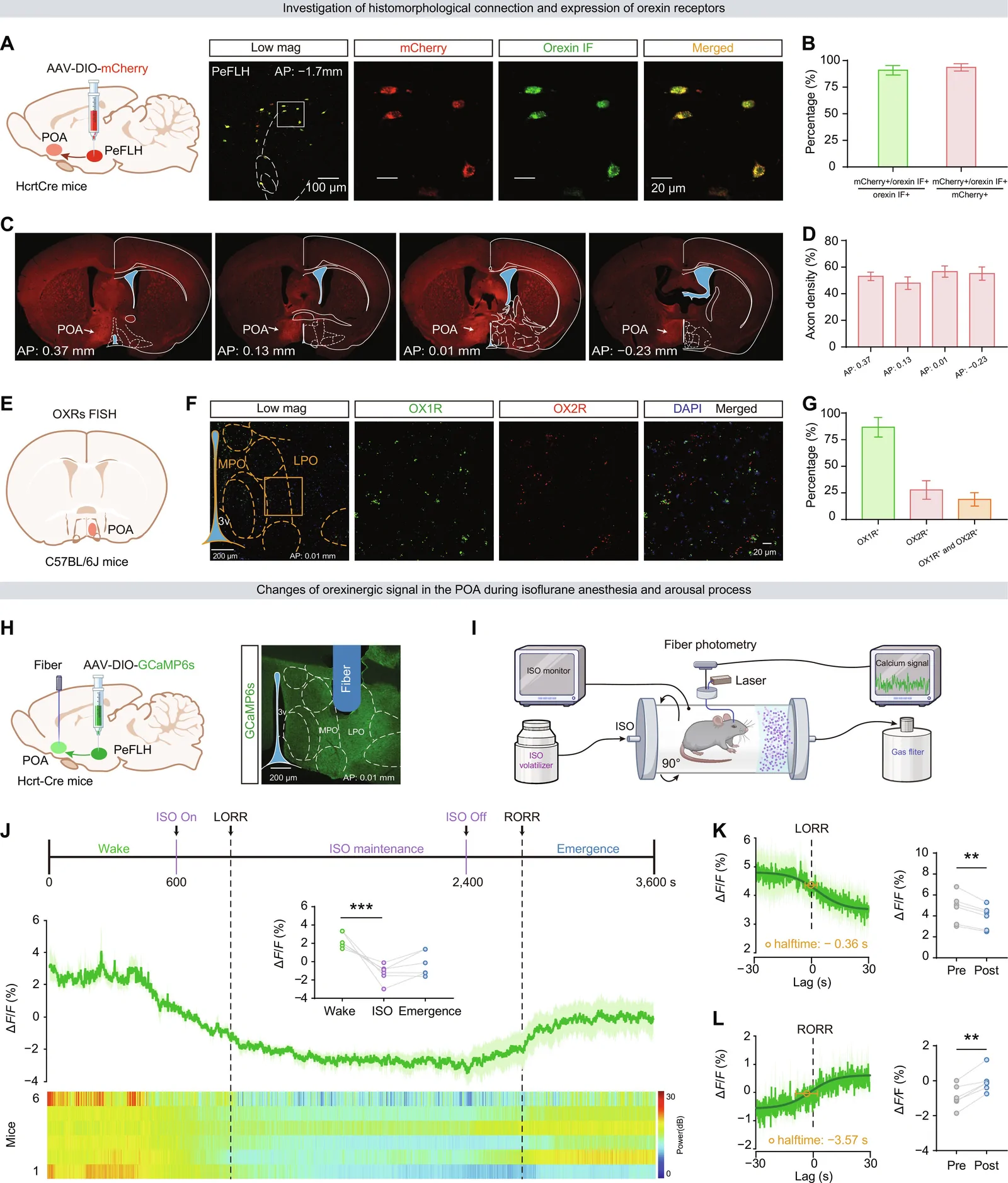
Changes of orexinergic signal in the preoptic area (POA) during isoflurane anesthesia and arousal process. (A) Left: Schematic of anterograde tracing. AAV-DIO-mCherry was injected into the perifornical lateral hypothalamus (PeFLH) in Hcrt-Cre mice. Right: Representative images showing co-localization of mCherry (red) with orexin-immunoreactive neurons (green) in the PeFLH. Scale bars: 100 μm (left), 20 μm (right). (B) Quantification of viral targeting efficiency, showing the percentage of mCherry-expressing neurons among all orexin-positive neurons. (C) Representative images showing dense mCherry-labeled orexinergic terminals in the POAat different anterior–posterior (AP) levels. (D) Quantification of orexinergic axon density at different AP levels of the POA. (E) Schematic illustrating the location of orexin receptor expression in the POA. (F and G) Representative fluorescence in situ hybridization (FISH) images and quantification of the expression of orexin-1 receptor (OX1R) and orexin-2 receptor (OX2R) in the POA. Cell nuclei were counterstained with 4′,6-diamidino-2-phenylindole (DAPI) (blue). (H) Left: Schematic of virus injection (AAV-DIO-GCaMP6s into PeFLH) and optic fiber implantation in the POA in Hcrt-Cre mice for fiber photometry. Right: Representative image of GCaMP6s expression and fiber placement in the POA. (I) Schematic of the in vivo fiber photometry recording setup. (J) Representative trace of GCaMP6s signals (Δ*F*/*F*, top) and corresponding heatmap (bottom) from an individual mouse during wakefulness, 1.4% isoflurane anesthesia (ISO), and emergence. Insets show expanded views of signal changes during loss of righting reflex (LORR) and recovery of righting reflex (RORR). Histogram in the middle: quantification of mean Δ*F*/*F* during wake, ISO, and emergence states. (K and L) Quantification of mean Δ*F*/*F* and fitting curve during LORR (K) and RORR (L) transitions, compared to the pre-transition baseline. IF, immunofluorescence; MPO, medial preoptic area; LPO, lateral preoptic area. **P < 0.01, ***P < 0.001. Statistical analysis: one-way ANOVA followed by Tukey post hoc test was used in (H), and paired *t* test was used in (H) and (I).

Having confirmed that orexinergic neurons strongly projected to the POA and orexin receptors expressed in the POA, we next assessed its physiological relevance. We used fiber photometry to measure calcium signal changes of orexinergic afferents in the POA during isoflurane general anesthesia and arousal process (Fig. 1H and I). The calcium signal intensity of POA orexin afferents was significantly suppressed upon administration of 1.4% isoflurane (Fig. 1J). Specifically, signal intensity decreased during loss of righting reflex (LORR) (Pre: 4.72 ± 1.42% vs. LORR: 3.88 ± 1.10%; *n* = 6, *P* = 0.0021) and increased during return of righting reflex (RORR) (Pre: −0.91 ± 0.66% vs. RORR: −0.04 ± 0.66%; *n* = 6, *P* = 0.0012) (Fig. 1K and L). Importantly, changes in POA orexinergic terminal calcium signal precede LORR (Lag: −0.36 [3.17] s) and RORR (Lag: −3.58 [5.69] s) (Fig. 1K and L). These results indicate that the activity of orexinergic projections in the POA is wake-state dependent and changes prior to consciousness state transitions induced by isoflurane anesthesia. These findings suggest orexinergic afferents in the POA as a physiologically relevant target whose activity dynamically predicts consciousness states during general anesthesia.

### Optogenetic modulation of POA orexinergic afferents causally controls arousal from isoflurane anesthesia

Based on the strong correlation observed, we next sought to determine a causal role for this pathway by using optogenetics (Fig. 2A to C). Cre-dependent virus with ChR2 were injected in the PeFLH and fibers were implanted in the POA in Hcrt-Cre mice. Following virus expression, excitatory (20 Hz 473 nm laser) and inhibitory (1 Hz 594 nm laser) were delivered via the fiber. We found that modulating this pathway bidirectionally controlled arousal from anesthesia. Optogenetic activation of orexinergic terminals in the POA significantly shortened the emergence time (ChR2: 375.0 [75.6] s vs. mCherry: 713.0 [135.2] s; *n* = 6, *P* = 0.0003) but did not influence the induction time (ChR2: 423.7 [22.2] s vs. mCherry: 423.0 [37.5] s; *n* = 6, *P* = 0.9709) (Fig. 2D). Conversely, inhibition of orexinergic afferents in the POA by expressing *Natronomonas pharaonis* halorhodopsin (NPHR), an inhibitory optogenetic component, did not influence the time to induction (NPHR: 439.2 [41.6] s vs. mCherry: 440.5 [47.4] s; *n* = 6, *P* = 0.9598), but increased the time to emergence (NPHR: 908.7 [75.2] s vs. mCherry: 744.2 [144.5] s; *n* = 6, *P* = 0.0329) (Fig. 2E). These results indicate that POA orexinergic afferents are sufficient and necessary for the regulation of behavioral arousal from anesthesia.

**Fig. 2.**
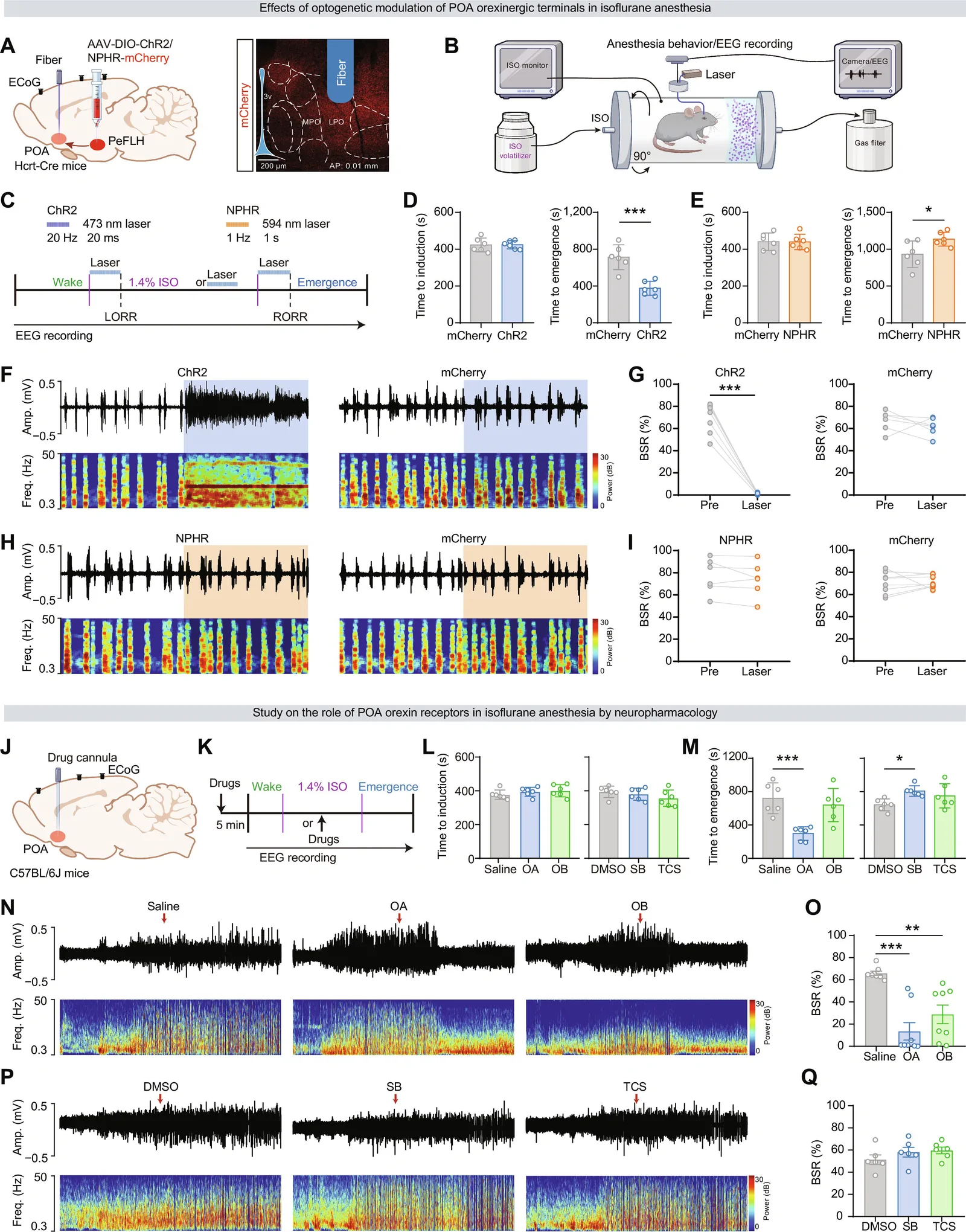
Optogenetic modulation of orexinergic terminals in the POA bidirectionally controls arousal from isoflurane anesthesia. (A) Left: Schematic of virus injection and optic fiber implantation. Right: Representative image of fiber placement and viral expression in the POA. (B) Schematic of the in vivo monitoring and anesthesia behavioral setup. (C) Experimental timeline for optogenetic stimulation (ChR2) or inhibition (NPHR). (D) Optogenetic activation of orexinergic terminals (ChR2) had no effect on induction time but significantly shortened emergence time compared to the mCherry control group. (E) Optogenetic inhibition (NPHR) had no effect on induction time but significantly prolonged emergence time. (F) Representative electrocorticography (ECoG) traces and corresponding spectrograms during anesthesia with (ChR2) and without (mCherry) optogenetic activation. (G) Quantification of burst-suppression ratio (BSR) before and during laser stimulation in ChR2 (left) and mCherry (right) mice. (H) Representative ECoG traces and corresponding spectrograms during anesthesia with (NPHR) and without (mCherry) optogenetic inhibition. (I) Quantification of BSR before and during laser stimulation in NPHR (left) and mCherry (right) mice. (J) Schematic of guide cannula implantation for bilateral microinjections into the POA. (K) Experimental timeline for microinjection of orexin agonists or antagonists. (L) Effects of intra-POA microinjection of orexin peptides and orexin receptor antagonists on the induction time, compared to saline controls. (M) Effects of intra-POA microinjection of orexin peptides and orexin receptor antagonists on the emergence time, compared to saline controls. (N) Representative raw ECoG traces and corresponding spectrograms during stable isoflurane anesthesia following intra-POA injection of saline, OA, or OB. (O) Quantification of the BSR following microinjection of saline, OA, and OB. (P) Representative raw ECoG traces and spectrograms following intra-POA injection of dimethyl sulfoxide (DMSO), SB, or TCS. (Q) Quantification of BSR following microinjection of DMSO, SB, and TCS. EEG, electroencephalogram; NPHR, *Natronomonas pharaonis* halorhodopsin. *P < 0.05, **P < 0.01, ***P < 0.001. Statistical analysis: unpaired *t* test was used in (D) and (E). Paired *t* test was used in (J) and (L). One-way ANOVA followed by Dunnett post hoc test was used in (L) and (M). One-way ANOVA followed by Tukey post hoc test was used in (N) and (O).

This causal role in behavior was mirrored by changes in brain state, as measured by ECoG (Fig. 2F to I). We found that optogenetic activation of POA orexinergic projections significantly reduced the burst-suppression ratio (BSR) in ECoG (ChR2: Pre: 71.72 [4.37] % vs. Laser: 1.18 [0.26] %; *n* = 9, *P* < 0.001), shifting the brain state toward arousal, but not in the control group (Fig. 2F and G). However, optogenetic inhibition of the orexinergic afferents in the POA could not affect the BSR in ECoG (NPHR: Pre: 77.07 [6.40] % vs. Laser: 73.86 [6.34] %; *n* = 6, *P* = 0.2736; Fig. 2H and I), likely because the BSR was already near maximum during deep anesthesia. These results suggest that activation of orexinergic afferents in the POA can accelerate and inhibition of which can delay emergence of consciousness from isoflurane general anesthesia, establishing that activity of orexin in the POA is a key target for modulating consciousness state in general anesthesia.

### Orexin-1 receptors primarily mediate the effect of orexinergic afferents in the POA

Given that both receptors are present, we sought to identify which one mediates the arousal-promoting effect. We first tested the effect of microinjecting orexin peptides and antagonists on anesthesia behavior and ECoG (Fig. 2J and K). We found that the time to induction did not influence by microinjection of orexin orexin-A and orexin-B (orexin-A: 394.2 [26.8] s vs. saline: 376.8 [28.1] s; *P* = 0.5278. orexin-B: 400.3 [35.85] s vs. saline: 376.8 [28.1] s; *P* = 0.3326. *n* = 6; Fig. 2L). However, microinjection of orexin-A in the POA significantly shortened the time to emergence, but orexin-B did not (orexin-A: 297.7 [80.71] s vs. saline: 720.6 [184.9] s; *P* < 0.001. orexin-B: 639.1 [199.3] s vs. saline: 720.6 [184.9] s; *P* = 0.4105. *n* = 6; Fig. 2M). This stronger effect of orexin-A, a potent agonist for both receptors, compared to the orexin-2 receptor (OX2R)-preferential orexin-B, provides initial evidence for orexin-1 receptor (OX1R) involvement. We further confirmed this using SB334867 (SB, OX1R antagonists) and TCS OX2-29 (TCS, OX2R antagonists), which also did not affect induction time (SB: 380.0 [35.8] s vs. dimethyl sulfoxide, DMSO: 393.8 [33.7] s; *P* = 0.7766. TCS: 356.3 [48.6] s vs. DMSO: 393.8 [33.70] s; *P* = 0.2125. *n* = 6; Fig. 2L). Crucially, microinjection of the OX1R antagonist SB334867, but not the OX2R antagonist TCS OX2-29, in the POA prolonged the time to emergence (SB: 807.1 [62.9] s vs. DMSO: 642.1 [70.67] s; *P* = 0.0224. TCS: 749.8 [145.6] s vs. DMSO: 642.1 [70.67] s; *P* = 0.1511. *n* = 6; Fig. 2M). These results indicate that POA orexin receptors are sufficient and necessary for the regulation of behavioral arousal during anesthesia.

These behavioral findings were corroborated by ECoG analysis. The schematic of the experimental setup is shown in Fig. 2J and K. We found that microinjection of orexin-A significantly reduced BSR in ECoG during isoflurane anesthesia maintenance, and orexin-B showed a weaker effect than orexin-A (orexin-A: 13.44 [7.84] % vs. saline: 65.91 [1.89] %; *P* < 0.001. orexin-B: 28.83% [8.39%] vs. saline: 65.91 [1.89] %; *P* = 0.0023. *n* = 8; Fig. 2N and O). Microinjection of either the OX1R antagonist or the OX2R antagonist failed to alter the BSR in ECoG during isoflurane anesthesia maintenance (Fig. 2P and Q). Taken together, these results indicate that while both OX1R and OX2R are expressed in the POA, the arousal-promoting effect of orexinergic input is mediated primarily by orexin-A acting on OX1R.

### Orexin peptides elicit bidirectional, heterogeneous responses in POA GABAergic neurons in vitro

Having defined the circuit-level function and molecular mediators, we next investigated the cellular substrate. Our results of multiplex fluorescent in situ hybridization revealed that 71.38% [1.7%] of POA GABAergic neurons express the orexin-1 receptor and 26.08% [3.6%] express the orexin-2 receptor (Fig. 3A and B). In POA glutamatergic neurons, 52.18% [6.9%] express the orexin-1 receptor and 19.93% [3.2%] express the orexin-2 receptor (Fig. 3C and D). This focused our investigation onto POA GABAergic neurons. To determine their intrinsic response to orexin, we examined whether and how orexin peptides act on GABAergic neurons in the POA. POA GABAergic neurons were patch clamped, the membrane potential (MP) and action potential (AP) were recorded (Fig. S1A and B). Unexpectedly, orexin peptides did not produce a uniform response; instead, they elicited heterogeneous, bidirectional modulation. With orexin-A (1 μM) bath applied, the firing activity of GABAergic neurons was either facilitated or inhibited (Fig. 3E). Eight of 15 GABAergic neurons were excited by orexin-A (OA), the AP firing frequency increased from 0.38 to 1.4 Hz (OA: 1.4 [1.14] Hz vs. Baseline: 0.38 [0.28] Hz; *n* = 8 cells from 3 mice, *P* = 0.0406), and the MP depolarized from −55.83 to −51.08 mV (OA: −51.08 [5.94] mV vs. Baseline: −55.83 [5.06] mV; *n* = 8 cells from 3 mice, *P* = 0.004) (Fig. 3F and H). Seven of 15 neurons were inhibited by orexin-A, the firing frequency was decreased from 0.28 to 0.002 Hz (OA: 0.0019 [0.005] Hz vs. Baseline: 0.28 [0.15] Hz; *n* = 7 cells from 3 mice, *P* = 0.0077), and the MP hyperpolarized from −55.45 to −61.51 mV (OA: −61.51 (6.977) mV vs. Baseline: −55.45 [4.691] mV; *n* = 7 cells from 3 mice, *P* = 0.0244) (Fig. 3G and H). Orexin-B application produced a similar, though slightly different, heterogeneous pattern (Fig. 3I). Six of 16 GABAergic neurons were excited by orexin-B (OB) and the firing frequency increased from 0.6 to 1.5 Hz (OB: 1.53 [0.79] Hz vs. Baseline: 0.60 [0.54] Hz; *n* = 6 cells from 4 mice, *P* = 0.0258) while the MP depolarized from −51.88 to −46.73 mV (OB: −46.73 [7.77] mV vs. Baseline: −51.88 [8.64] mV; *n* = 6 cells from 4 mice, *P* = 0.0005) (Fig. 3J and L). Five of 16 GABAergic neurons were inhibited by orexin-B (OB: 0.03 [0.048] Hz vs. Baseline: 0.45 [0.43] Hz; *n* = 5 cells from 4 mice, *P* = 0.0294) and the MP hyperpolarized from −54.84 to −60.58 mV (OB: −60.58 [6.30] mV vs. Baseline: −54.84 [3.57] mV; *n* = 5 cells from 4 mice, *P* = 0.0336) (Fig. 3K and L). Five of 16 GABAergic neurons were not responsive to orexin-B (Fig. 3L). These data indicate that orexin-A and orexin-B are both capable of exciting or inhibiting POA GABAergic neurons in vitro, revealing a complex cellular response rather than simple suppression.

**Fig. 3.**
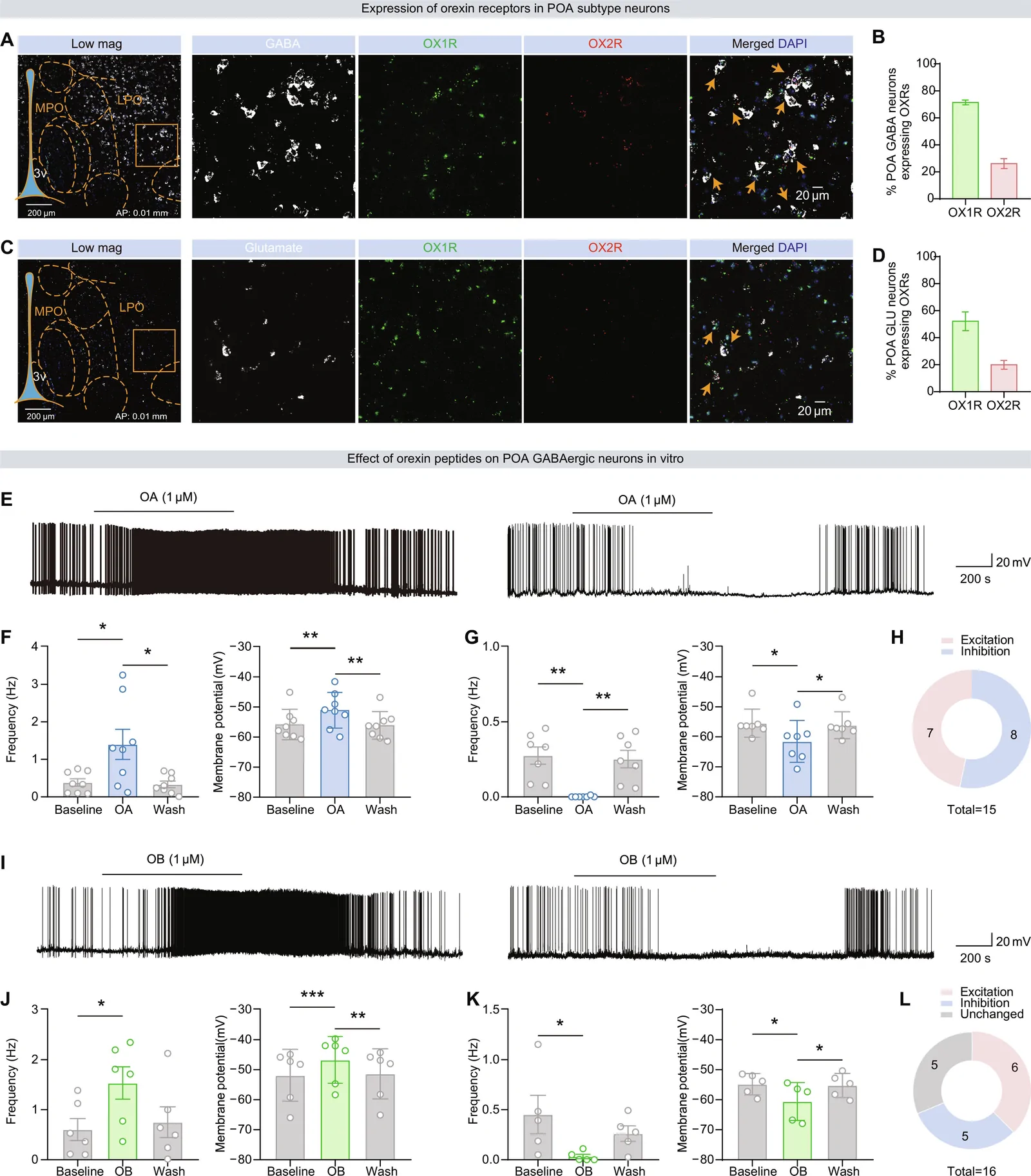
Orexin peptides bidirectionally modulate the activity of POA GABAergic neurons in vitro. (A and B) Representative fluorescence in situ hybridization images and quantification of the expression of OX1R and OX2R in GABAergic neurons in the POA. Cell nuclei were counterstained with DAPI (blue). (C and D) Representative fluorescence in situ hybridization images and quantification of the expression of OX1R and OX2R in glutamate neurons in the POA. Cell nuclei were counterstained with DAPI (blue). (E) Representative traces showing heterogeneous responses of 2 different POA GABAergic neurons to bath application of orexin-A (OA, 1 μM). One neuron was excited (left) while the other was inhibited (right). (F) Quantification of action potential frequency and resting membrane potential in OA-excited neurons before (Baseline), during (OA), and after (Wash) drug application. (G) Quantification of action potential frequency and resting membrane potential in OA-inhibited neurons. (H) Proportion of recorded POA GABAergic neurons that were excited or inhibited by OA (*n* = 15 cells from 4 mice). (I) Representative traces showing heterogeneous responses of 2 different POA GABAergic neurons to bath application of orexin-B (OB, 1 μM). One neuron was excited (left) while the other was inhibited (right). (J) Quantification of action potential frequency and resting membrane potential in orexin-B (OB, 1 μM)-excited neurons. (K) Quantification of action potential frequency and resting membrane potential in OB-inhibited neurons. (L) Proportion of recorded POA GABAergic neurons that were excited, inhibited, or nonresponsive to OB (*n* = 16 cells from 4 mice). *P < 0.05, **P < 0.01, ***P < 0.001. Statistical analysis: one-way ANOVA followed by Tukey post hoc test was used in (F), (G), (J), and (K).

### Isoflurane anesthesia suppresses the firing of POA GABAergic neurons and alters communication between POA and the frontal cortex

Our in vitro data revealed a surprising heterogeneity. We next sought to determine if this complex modulation also occurs in the intact, anesthetized animal. To do this, we first characterized the baseline activity of POA neurons during anesthesia. In this step, we took advantage of an opto-tetrode with double-virus systems in Hcrt-Cre mice, in which a virus expressing NPHR with the Vgat1 promoter was used to target POA GABAergic neurons and a Cre-dependent virus expressing ChR2 was used to target orexin neurons (Fig. 4A and B). This strategy allowed us to record the firing of GABAergic neurons while simultaneously enabling the optogenetic activation of orexinergic terminals. As a first step, 92 of 124 POA neuronal firings were identified by their firing suppression (sample data, Pre: 3.79 [1.22] spikes/s vs. Laser: 0.27 [0.07] spikes/s, *n* = 13, *P* = 0.0307) in response to yellow-light illumination, which activated NPHR (Fig. 4C). Representative extracellular waveforms of these optogenetically identified POA GABAergic neurons are shown in Fig. 4B, with AP durations ranging from approximately 0.45 to 0.61 ms and approximately 5% of the recorded spikes occurring in bursts.

**Fig. 4.**
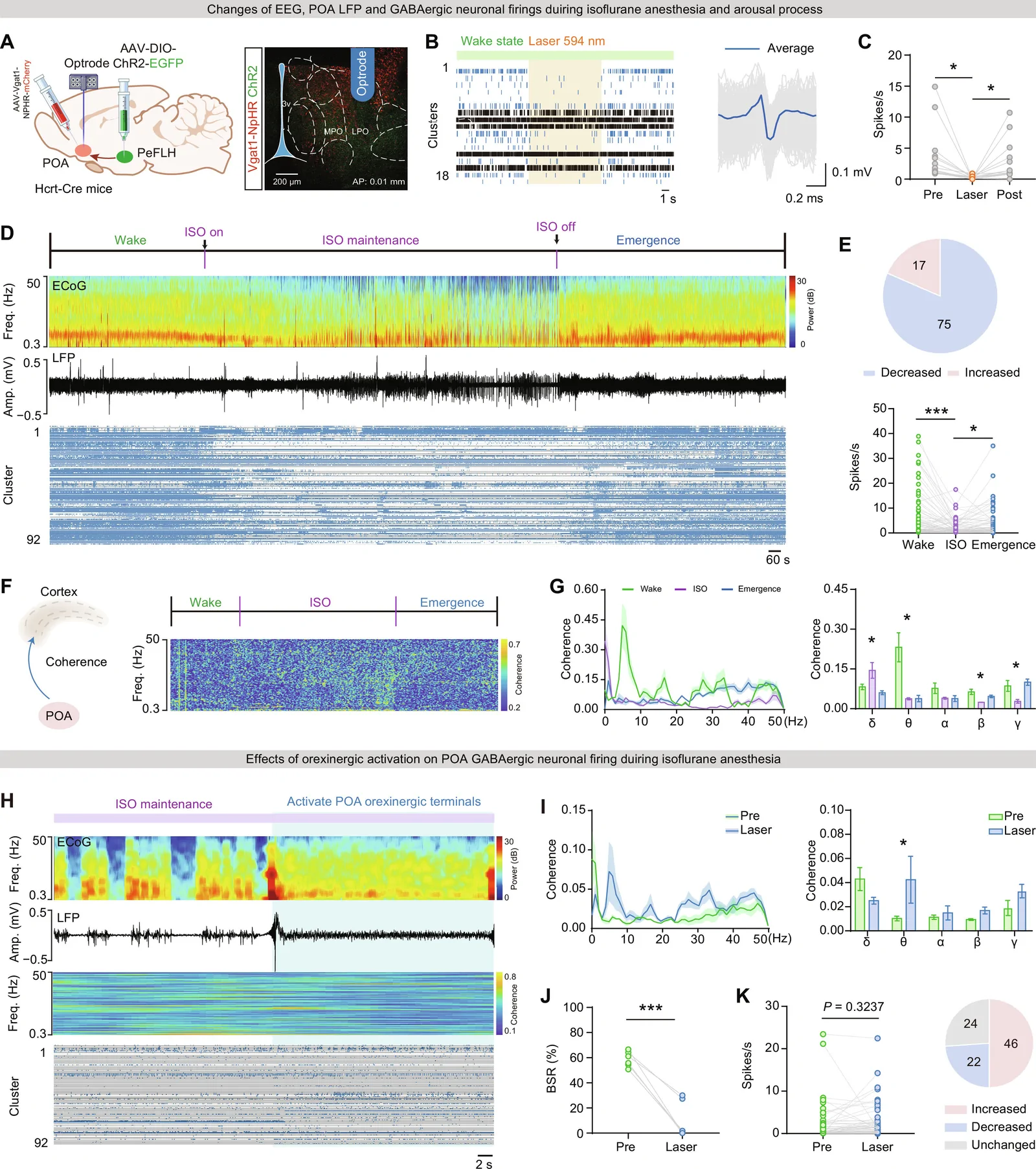
Orexinergic activation elicits heterogeneous responses from POA GABAergic neurons in vivo. (A) Left: Schematic of the in vivo opto-tetrode recording setup. A dual-virus strategy was employed, enabling both the optogenetic identification of POA GABAergic neurons and the activation of orexinergic terminals. Right: Representative fluorescence image showing the electrode track and viral expression in the POA. (B) Identification of GABAergic neurons. Left: A representative neuron’s firing activity is suppressed during 594-nm laser stimulation, as shown in the raster plot, representative auto-correlogram and waveform. (C) Quantification of firing rates before, during, and after optogenetic inhibition, confirming successful identification. (D) Representative local field potential (LFP) spectrogram (top) and population raster plot (bottom) of simultaneously recorded POA neurons across Wake, 1.4% isoflurane anesthesia (ISO), and Emergence states. (E) Quantification of average firing rates across the 3 states, showing a significant reduction during anesthesia. (F) Schematic of coherence analysis between POA and frontal cortex ECoG and representative heatmap during anesthesia and arousal process. (G) Left: Coherence traces between POA and frontal cortex ECoG in wake, ISO, and emergence states. Right: Quantification of coherence in distinct frequency bands. (H) Representative recording during isoflurane anesthesia. Top: ECoG spectrogram, raw LFP trace, and coherence heatmap showing the effect of optogenetic activation of orexinergic terminals (blue shaded area). Bottom: Raster plot of simultaneously recorded POA GABAergic neuronal firing. (I) Left: Coherence traces between POA and frontal cortex ECoG in wake, ISO, and emergence states. Right: Quantification of coherence in distinct frequency bands. (J) Quantification of the BSR, which was significantly reduced by the stimulation. (K) Left: Population firing rates (spikes/s) remained unchanged on average before and during stimulation. Right: Pie chart illustrating the heterogeneous cellular responses among the 92 recorded neurons. *P < 0.05, ***P < 0.001. Statistical analysis: one-way ANOVA followed by Tukey post hoc test was used in (C) and (E). Two-way ANOVA followed by Bonferroni multiple comparisons test was used in (G) and (I). Paired *t* test was used in (J) and (K).

Next, we performed the isoflurane anesthesia and arousal process (Fig. 4D). The firing rates of POA GABAergic neurons mainly decreased during isoflurane anesthesia (ISO) (Wake: 5.47 [0.91] spikes/s vs. ISO: 1.72 [0.29] spikes/s, *n* = 92, *P* < 0.001), although 17 neurons exhibited elevated activity (Fig. 4E). To rule out potential sex-specific bias in these firing patterns, we next performed an exploratory sex-stratified analysis of POA GABAergic neuronal firing across wakefulness, isoflurane anesthesia, and emergence. Although neuronal firing varied significantly across consciousness states, no significant main effect of sex or sex-by-state interaction was observed (Fig. S2A and B), indicating that no evident sex-specific differences were detected under the present experimental conditions. Moreover, local field potential (LFP) in the POA showed a delta-band increase (Wake: 27.15 [1.82] % vs. ISO: 42.50 [1.79] %, *n* = 6, *P* = 0.0028) and theta-band decrease (Wake: 43.75 [1.17] % vs. ISO: 30.11 [1.17] %, *n* = 6, *P* = 0.0028) during isoflurane anesthesia (Fig. S3A). The ECoG showed similar changes as LFP in the POA during isoflurane general anesthesia and arousal process (Fig. S3B). Furthermore, the coherence changes, which indicate the communication alternation between POA and the frontal cortex, were analyzed (Fig. 4F). We found that isoflurane anesthesia significantly increased the delta-band coherence between POA and the frontal cortex (ISO: 0.159 [0.075] vs. Wake: 0.084 [0.03], *n* = 6, *P* = 0.0199; Fig. 4G) and decreased the theta-, beta-, and gamma-band coherence. These results suggest that both communication between the POA and the frontal cortex and POA GABAergic neuronal firings were inhibited during isoflurane anesthesia, indicating that POA is key to arousal from general anesthesia.

### POA GABAergic neurons exhibit heterogeneous firing responses to orexinergic activation in vivo

To determine how orexinergic inputs reshape POA microcircuit activity under anesthesia, we optogenetically activated orexinergic terminals in the POA during isoflurane general anesthesia maintenance while simultaneously recording GABAergic neuronal firing, LFP, and ECoG (Fig. 4H). Optical stimulation induced a robust shift toward an aroused state: It significantly suppressed δ (delta) power while enhancing high-frequency (α, β, and γ) power in both local LFP and cortical ECoG (Fig. S3C and D). Concurrently, θ (theta) band coherence between the POA and frontal cortex was significantly increased (Fig. 4I), and the BSR dropped markedly (Pre: 58.86 ± 6.13% vs. Laser: 10.16 ± 14.42%; *n* = 6 mice, *P* < 0.001; Fig. 4J). Despite these electrophysiological changes, no overt arousal-related behavioral changes, such as body movement, were observed during optogenetic stimulation.

At the single-unit level, although the mean population firing rate of POA GABAergic neurons showed a nonsignificant trend toward increase (Pre: 1.36 ± 0.37 vs. Laser: 1.68 ± 0.35 spikes/s; *P* = 0.3237; Fig. 4K), this aggregate metric masked a striking functional heterogeneity. Specifically, optical activation bidirectionally modulated neuronal activity: 46 of 92 recorded neurons (50%) exhibited increased firing rates, whereas 22 (24%) were suppressed, and the remainder were unchanged (Fig. 4K). This divergence suggests that orexin does not uniformly excite the POA network; rather, it likely selectively recruits specific subpopulations to orchestrate the observed network-level desynchronization and arousal, a hypothesis we next tested by identifying cell-type-specific responses.

### POA parvalbumin neuronal firings mediated feedforward inhibition responses to orexinergic activation in vivo

Previous studies have indicated that parvalbumin (PV)-positive neurons are expressed in the POA and play an important role in microcircuit regulation [16,17]. First, we examined the expression of orexin receptors on POA PV-positive neurons. In situ hybridization results showed that 85.53% [2.65%] of POA GABAergic neurons express the orexin-1 receptor and 44.30% [3.16%] express the orexin-2 receptor (Fig. 5A and B). Given that these PV neurons probably are GABAergic interneurons, they are anatomically positioned to mediate local inhibition within the POA microcircuit. To investigate the possible microcircuit mechanism of POA mediating orexinergic activation, we targeted PV-positive neurons by a double-virus system and in vivo electrophysiology. In detail, a PV promoter with NPHR was injected into the POA, Cre-dependent ChR2 was injected into the PeFLH, and a 16-channel opto-tetrode was implanted in the POA (Fig. 5C). Following the identification of putative PV-positive (PV^+^) neuronal firings via optogenetic inhibition during the wake state (Fig. 5D), their role in the POA microcircuit was analyzed. We found that PV-negative (PV^−^) GABAergic neuronal firings exhibited a significant increase during inhibition of PV^+^ neuronal firings (Pre: 2.79 [0.61] spikes/s vs. Laser: 4.36 [0.8] spikes/s, *n* = 28, *P* = 0.0127; Fig. 5E). Importantly, the changes of PV^+^ neuronal firings preceded PV^−^ neuronal firings when inhibitory laser was performed (PV^+^: 38.18 [93.31] ms vs. PV^−^: 223.9 [188.0] ms, *P* = 0.0352; Fig. 5F). These findings are consistent with an inhibitory influence of a subset of PV-positive neurons on other POA GABAergic neurons, although they do not establish a direct monosynaptic connection.

**Fig. 5.**
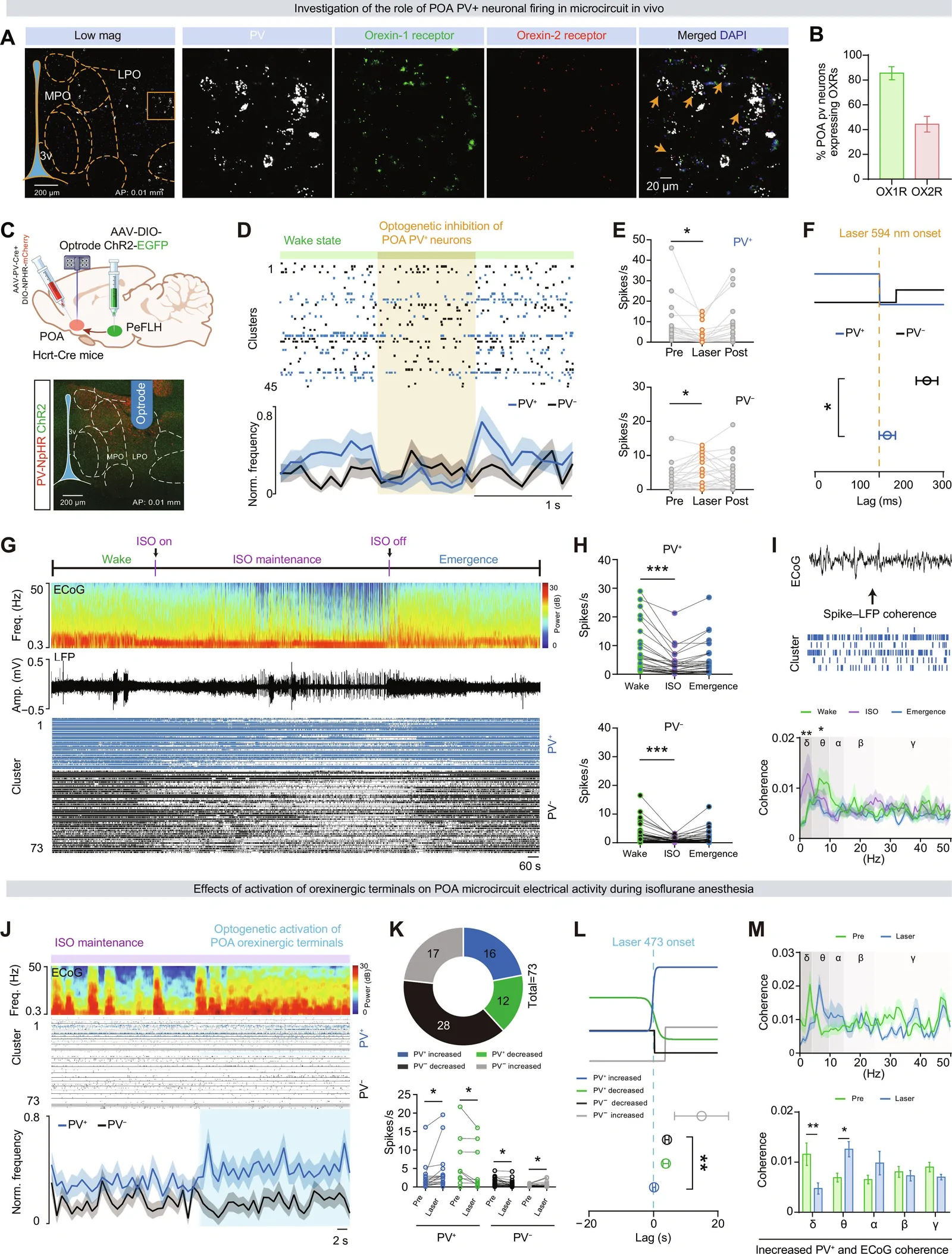
Orexinergic activation induced parvalbumin neuronal firings mediated feedforward inhibition in the POA. (A and B) Representative fluorescence in situ hybridization images and quantification of the expression of OX1R and OX2R in parvalbumin neurons in the POA. Cell nuclei were counterstained with DAPI (blue). (C) Schematic of the experimental strategy using an opto-tetrode and a dual-virus system to record from photo-identified PV neurons while stimulating orexin terminals (top). Representative image shows the dual-virus expression and cannula. (D) Identification of a putative parvalbumin-positive (PV^+^) neuron via optogenetic inhibition. (E) Quantification of rates for photo-identified PV^+^ and PV-negative (PV^−^) neuronal firing during laser inhibition. (F) Quantification of changes of PV^+^ and PV^−^ neuronal firing and fitting curve during laser inhibition. (G) Representative ECoG spectrogram, LFP raw trace (top), and population raster plot (bottom) of simultaneously recorded POA PV^+^ and PV^−^ neuronal firings across 3 different states. (H) Quantification of average PV^+^ and PV^−^ neuronal firing rates across the 3 states, both showing a significant reduction during anesthesia. (I) Schematic (top) and quantification (bottom) of POA spike and ECoG coherence in 3 different states. (J) Representative ECoG spectrogram, LFP raw trace, and raster plot during isoflurane anesthesia maintenance with optogenetic activation. (K) Quantification of rates for PV^+^ and PV^−^ neuronal firings before and during orexinergic activation in the isoflurane anesthesia state. (L) Quantification of changes of PV^+^ and PV^−^ neuronal firing and fitting curve before and during orexinergic activation in the isoflurane anesthesia state. (M) Traces and quantification of coherence of increased POA PV^+^ neuronal firing and frontal cortex ECoG during optogenetic activation orexinergic terminals. *P < 0.05, **P < 0.01, ***P < 0.001. Statistical analysis: one-way ANOVA followed by Bonferroni multiple comparisons test was used in (E) and (H). Unpaired *t* test was used in (F) and (L). Paired *t* test was used in (K). Two-way ANOVA followed by Bonferroni multiple comparisons test was used in (I) and (M).

Next, we characterized how POA PV^+^ and PV^−^ neuronal firings respond to isoflurane anesthesia (Fig. 5G). We found that the transition from wakefulness to isoflurane anesthesia significantly suppressed the firing rates of both the identified PV^+^ neurons (Wake: 8.22 [1.55] spikes/s vs. ISO: 3.55 [0.85] spikes/s, *n* = 28, *P* < 0.001) and the neighboring PV^−^ GABAergic neurons (Wake: 2.64 [0.48] spikes/s vs. ISO: 0.79 [0.12] spikes/s, *n* = 45, *P* < 0.001) (Fig. 5H). To further examine the relationship between POA PV^+^ neuronal activity and cortical states, we analyzed the coherence between optogenetically identified PV^+^ and PV^−^ neuronal firing and simultaneously recorded frontal cortical ECoG activity during isoflurane anesthesia and arousal process. During wakefulness, PV^+^ neuronal firing showed the highest coherence with the theta band, whereas during isoflurane anesthesia the highest coherence was observed in the delta band (Fig. 5I and Fig. S4A). Furthermore, the gamma-band coherence of PV^−^ neuronal firing and cortical activity was increased during isoflurane anesthesia (Fig. S4B).

To study how orexinergic afferents modulate the POA microcircuit, we then proceeded to investigate the effect of orexinergic terminal activation on these two neuronal populations during anesthesia (Fig. 5J). Subsequently, the firing rate changes of these PV^+^ and PV^−^ neuronal firings before and during orexinergic activation were analyzed. We found that during isoflurane anesthesia maintenance, 16 of 28 PV^+^ neuronal firings exhibited an increase (Pre: 2.55 [0.97] spikes/s vs. Laser: 4.34 [1.38] spikes/s, *n* = 16, *P* = 0.0367) and 12 of 28 PV^+^ neuronal firings exhibited a decrease (Pre: 4.91 [1.93] spikes/s vs. Laser: 3.7 [1.65] spikes/s, *n* = 6, *P* = 0.0495) in response to orexinergic activation (Fig. 5K). Meanwhile, 28 of 45 PV^−^ neuronal firings exhibited a decrease (Pre: 1.05 [0.21] spikes/s vs. Laser: 0.71 [0.18] spikes/s, *n* = 28, *P* = 0.0158) and 17 of 45 PV^−^ neuronal firings exhibited an increase (Pre: 0.3 [0.07] spikes/s vs. Laser: 0.63 [0.19] spikes/s, *n* = 17, *P* = 0.0185) in response to orexinergic activation (Fig. 5K). Thus, neither the PV-positive nor the PV-negative neuronal population showed a uniform response to orexinergic terminal activation. More importantly, the changes of increased PV^+^ neuronal firings preceded decreased PV^−^ neuronal firings (PV^+^ increased: 0.2 [0.2] s vs. PV^−^ decreased: 1.91 [0.26] s, *P* = 0.0401) and decreased PV^+^ neuronal firings (Fig. 5L). Interestingly, the increased PV^−^ neuronal firings changed 13 [2.317] s following orexinergic activation (Fig. 5L). Also, we found that optogenetic activation of orexinergic terminals in the POA during isoflurane anesthesia increased the theta-band coherence of PV^+^ neuronal firing and ECoG (Pre: 0.0069 [0.0034] vs. Laser: 0.0125 [0.0061], *n* = 16, *P* = 0.0213; Fig. 5M). Together, these temporal dynamics are consistent with a putative feedforward inhibitory process involving specific orexin-excited PV-positive and orexin-inhibited PV-negative neuronal subgroups.

## Discussion

This study provides direct electrophysiological evidence for a key mechanism whereby the neuronal firing within the POA microcircuit dynamically responds to orexin-induced arousal from isoflurane general anesthesia. We established solid evidence, including changes in orexinergic terminal activity in the POA that precede conscious-state transition, up-regulation of POA orexinergic afferents that causally induces and promotes the emergence of consciousness from anesthesia, and the primary mediation of this effect by orexin-A signaling through OX1R. Critically, our central finding is that this orexin-induced effect is not achieved by a simple, uniform influence on the POA microcircuit. Instead, it is implemented via a complex, heterogeneous modulation of POA GABAergic neurons, in which specific PV-positive and PV-negative neuronal subgroups may contribute to orexin-induced arousal through a feedforward inhibitory process (Fig. 6).

**Fig. 6.**
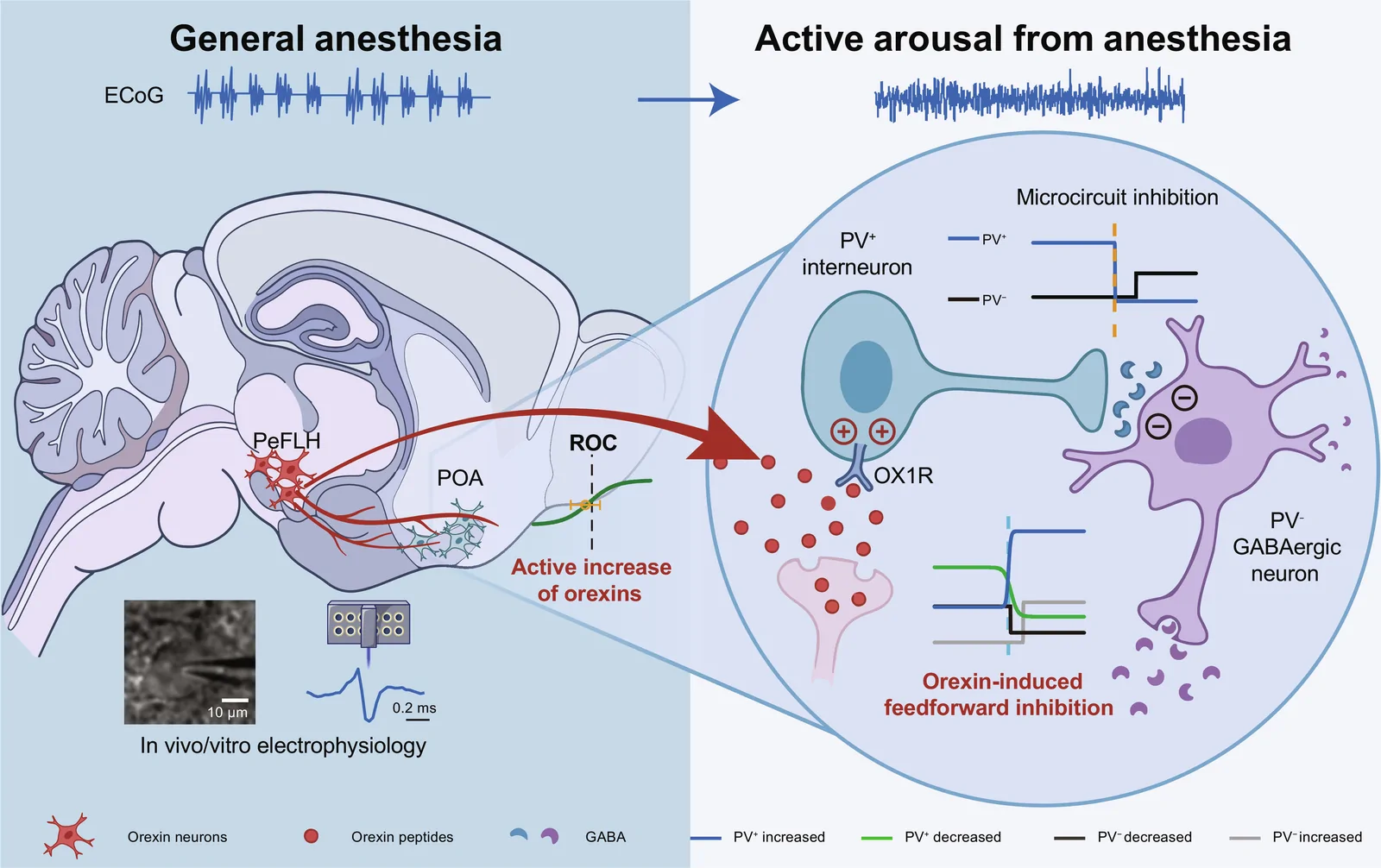
Graphical abstract. Schematic indicates that the increase of orexin peptides in the POA precedes consciousness recovery from general anesthesia, actively promoting arousal through a PV-neuron-mediated disinhibition mechanism. ECoG, electrocorticography; PV, parvalbumin; PeFLH, perifornical lateral hypothalamus; POA, preoptic area; ROC, recovery of consciousness; OX1R, orexin-1 receptor.

Our findings reveal a novel, parallel pathway for orexin-mediated arousal that complements the classical model. Indeed, the orexinergic system is well known to promote arousal by directly exciting the ascending reticular activating system, including the locus coeruleus and VTA [11,18,19]. We found that, simultaneously, orexin uses a more sophisticated “push-pull” strategy: it not only “pushes” wake-promoting ascending reticular activating system nuclei but also “pulls”, or more accurately, “reconfigures”, the sleep-promoting POA. Such a 2-front strategy likely underlies a more robust and efficient transition from the deeply suppressed anesthetic state to consciousness [17]. Importantly, we demonstrated that activity changes in POA orexinergic terminals precede conscious-state transitions. Although the decrease in the calcium signal intensity of orexinergic terminals in the POA preceded isoflurane-induced loss of righting reflex by approximately 0.36 s, optogenetic manipulation of these afferents did not significantly affect anesthesia induction. Therefore, the reduction in orexinergic terminal activity during induction may be involved in the regulation of behaviors not directly associated with arousal. In contrast, the bidirectional effects of orexinergic terminal manipulation on emergence suggest that the orexin system may play a “trigger” role during recovery from general anesthesia. This interpretation is consistent with our previous work and studies by others [20,21]. Our previous studies showed that plasma orexin-A concentrations in rats during the emergence state were higher than those before propofol and isoflurane anesthesia, whereas Fos expression in orexin neurons had not returned to the pre-isoflurane anesthesia level [10,19]. In the present study, during the 20-min emergence period, the calcium signal intensity of orexinergic terminals in the POA partially recovered from the anesthesia state but did not return to the pre-anesthesia level. This incomplete recovery may be related to incomplete metabolism of isoflurane in mice. Because the present study focused on the transient mechanisms underlying the loss and recovery of consciousness, future studies should extend the emergence observation period to determine whether orexinergic terminal activity in different brain regions returns to pre-anesthesia levels.

Mechanistically, our pharmacological data identify OX1R as the predominant player in the POA in mediating orexin-induced arousal. This is of particular interest in comparison with the regulation of natural sleep, in which OX2R is often considered to play a dominant role [18,22–24]. This divergence suggests that anesthetic emergence and natural awakening, while sharing pathways, may rely on distinct molecular mechanisms. Although our in vitro recordings show that both orexin-A and the OX2R-preferential orexin-B can modulate POA neurons, our in vivo data point to a clear dominance of OX1R in promoting emergence from anesthesia. Intra-POA administration of the OX1R antagonist significantly delayed emergence, whereas OX2R antagonism produced a trend toward delayed recovery that did not reach statistical significance. Although published in vitro studies suggest that SB334867 and TCS-OX2-29 have antagonist potencies within a broadly similar range at their respective receptors [25,26], equivalent receptor occupancy or blockade within the POA was not directly demonstrated. Therefore, the different pharmacological outcomes should be interpreted cautiously, and a potential contribution of OX2R cannot be excluded. Whether OX2R in the POA plays a role under other general anesthetics or in other consciousness-related behaviors remains to be further explored. This aligns with previous work from our group and others suggesting a key role for OX1R in anesthesia emergence [18,22,27]. This dominance of OX1R may be due to several potential factors. For instance, while both receptors are functional at the cellular level, the specific microcircuit activated by OX1R may be more critical for driving the network-level state transition required for anesthesia emergence. Our finding that both receptors are expressed is consistent with previous in situ hybridization studies [28]. This finding supports the potential for developing selective OX1R agonists as novel pro-emergence therapeutics, which may offer greater specificity and fewer side effects than general stimulants [29,30].

The most striking finding in our study is the heterogeneous response of POA GABAergic neurons to orexinergic input. Notably, although optogenetic activation of orexinergic terminals markedly altered ECoG features, no overt arousal-related behaviors, such as body movement, were observed during stimulation. This electrophysiological–behavioral dissociation may indicate that activation of orexinergic input to the POA alone was insufficient to fully reverse the unconscious state maintained at the anesthetic depth used. Moreover, the absence of a significant change in the overall population firing rate may have reflected opposing responses among distinct GABAergic neuronal subpopulations. Our targeting of PV-positive neurons provides a structural basis for this sophisticated microcircuit computation. The data are consistent with a feedforward inhibition model and heterogeneous neural circuits described in sleep research [17,31]. In this model, orexin may directly excite a population of local PV-positive GABAergic interneurons, which represent the fast excited neuronal firings we identified in our recordings. These interneurons, in turn, would then release GABA onto the primary sleep-promoting output neurons (likely represented by the PV-negative population), thus inhibiting them. Ultrastructural studies in cortical and striatal circuits have shown that GABAergic PV-positive interneurons commonly form symmetric inhibitory synapses, whereas many excitatory afferents onto PV-positive neurons form asymmetric synapses [32,33]. By analogy, POA PV-positive neurons may inhibit PV-negative neurons through Gray type II-like synaptic contacts, thereby contributing to the proposed feedforward inhibitory circuit [33]. However, this synaptic organization was not directly examined in the present study. Interestingly, we found that the increase in firing of orexin-excited PV-positive neurons preceded the decrease in firing of both PV-negative and orexin-inhibited PV-positive neurons. Furthermore, we also found that a cluster of PV-negative neuronal firings showed a delayed activation, which may be due to the whole brain achieving a wake-like state and interacting with multiple neural circuits. These findings indicate that orexinergic input elicits both excitatory and inhibitory responses within the POA microcircuit. Within the responsive subgroups, excitation of PV-positive neurons preceded inhibition of PV-negative neurons, providing temporal and functional evidence consistent with a putative PV-neuron-mediated feedforward inhibitory process during emergence from anesthesia. While our opto-tetrode recordings provide direct evidence for this local feedforward inhibition, we acknowledge the boundaries of this simplified model. First, orexin peptides can act via volume transmission, meaning their influence likely extends beyond the immediate release sites to distal receptor-expressing populations not fully captured by our localized recordings. Second, our study primarily focused on intra-POA local interactions; however, POA PV-positive neurons may also send long-range extra-POA projections to other arousal-related centers. Therefore, the PV-neuron-mediated microcircuit proposed here should be viewed as a critical local component—a precise computational reconfiguration within the POA—rather than the sole mechanism of orexin-induced arousal. This is critical, as previous studies have shown that lesioning the POA can facilitate emergence [34], while stimulation causes sleep [17,35], and the POA is known to contain a majority of GABAergic neurons [36] which regulate sleep [37].

Previous studies have indicated that effective information communication including feedback and feedforward between the cortex and subcortical regions is one of the essential conditions for consciousness [38,39]. We found that delta-band power in both POA LFP and frontal cortex ECoG was increased during isoflurane-anesthesia-induced unconsciousness state, while the higher bands were decreased. Based on the analysis of coherence between POA LFP and frontal cortex ECoG, we proved that the delta band dominates in POA–frontal cortex communication in general anesthesia. Interestingly, although much higher bands such as alpha and beta bands in POA LFP and ECoG were increased by orexinergic activation, the theta-band coherence between POA and the frontal cortex was significantly increased. These findings are partly consistent with those from our previous study, in which we showed that orexinergic activation increased theta- and alpha-band coherence between the nucleus accumbens and the frontal cortex [27]. Our results suggest that neural electrical activity communication between subcortical regions and the cortex is a potential target to monitor consciousness state during clinical general anesthesia.

Though our study revealed a robust functional connection, several limitations still remain. First, how orexin neuropeptides precisely regulate the electrical activity of different types of neurons in the POA remains unclear. Future approaches that simultaneously monitor neurotransmitter concentration changes and electrical activity are expected to address this question [36]. Second, although our data strongly support a microcircuit model involving PV neurons, there is still a great need to further characterize other potential subpopulations involved in this regulation—for example, by examining the interaction between PV and Galanin-positive neurons. This includes exploring the role of galanin co-transmission [31,40,41]. Finally, although our study included approximately balanced numbers of male and female mice, it was not sufficiently powered to systematically assess sex differences. Given that previous work has reported sex-dependent anesthetic sensitivity and subcortical activity patterns [42], potential sex-dependent effects on the orexin–POA circuit warrant further targeted investigations. In conclusion, further investigations regarding the functional importance of this pathway in postoperative and sleep disturbances and translating these findings into clinically viable drugs targeting OX1R will be important avenues for future research.

In conclusion, our findings support an important role for the sleep-promoting POA in orexin-mediated regulation of consciousness states during general anesthesia. Orexinergic input may facilitate emergence from isoflurane anesthesia by reorganizing heterogeneous GABAergic microcircuits within the POA rather than simply suppressing their overall activity. These findings provide insight into the neural mechanisms underlying anesthesia-related consciousness transitions and suggest that the orexin–POA pathway may represent a potential target for future modulation of anesthetic emergence.

## Methods

### Animals

Hcrt-Cre mice were supplied by Cyagen Biosciences (Suzhou, China) and C57BL/6J mice were obtained from the Vital River Experimental Animal Technology Co., Ltd. (Beijing, China). Mice were housed under specific pathogen-free conditions. The environment was maintained at a constant temperature (21 to 23 °C) and humidity (38% to 42%). The light cycle was controlled (lights on at 7:00 a.m. and off at 7:00 p.m.), and food and water were available ad libitum. Adult male and female mice (6 to 8 wk old) were used in approximately equal numbers (Table S1). Unless otherwise specified, data from both sexes were pooled for the primary analyses. An exploratory sex-stratified analysis was conducted to compare POA GABAergic neuronal firing across wakefulness, isoflurane anesthesia, and emergence. We performed all experimental procedures following the protocol approved by the Animal Experiment Ethics Committee and strictly adhering to the guidelines for animal experiments at the Fourth Medical University (Xi’an, China).

### Viral vectors and constructs

In fiber photometry experiments, AAV2/9-EF1α-DIO-GCaMP6s (PT-0071, 5.04E+12 vg/mL) were used. In optogenetic experiments, AAV2/9-EF1α-DIO-mCherry (PT-0013, 5.2E+12 vg/mL), AAV2/9-EF1α-DIO-ChR2-mCherry (PT-0002, 5.08E+12 vg/mL), and AAV2/9-EF1α-DIO-NPHR-mCherry (PT-0007, 5.02E+12 vg/ml) were used. AAV2/9-Vgat1-NPHR-mCherry (PT-9113, 5.13E+12 vg/mL), AAV2/9-PV-Cre (PT-0275, 5.23E+12 vg/m), and AAV2/9-EF1α-DIO-ChR2-EGFP (PT-9363, 5.22E+ 12 vg/mL) were used for the in vivo electrophysiological experiments. All of these viruses were provided by Brain VTA Technology Co., Ltd. (Wuhan, China).

### Stereotaxic surgery and implantation

#### Surgical procedures

Mice were anesthetized by 1.4% isoflurane in 100% oxygen (flow rate: 1.0 L/min) in an induction cage. Then, we fixed the head of mice on a stereotaxic frame. Isoflurane (0.8% to 1.2%) delivered through a mask was used for anesthesia maintenance. For local analgesia, 0.5 mL of 1% lidocaine was administered subcutaneously at the incision site at both the beginning and end of the stereotaxic surgery. Meanwhile, a heating pad which were set at 37 °C were used to maintain the body temperatures of mice. Hydrogen peroxide (3%) was used to remove the fascia from the skull surface following disinfection of the incision site with iodine. The mouse’s head was adjusted to a horizontal position using the bregma point as a reference.

#### Establishment of an optogenetic and fiber photometry model

All viruses were microinjected at a flow rate set at 50 nL/min. AAV2/9-EF1α-DIO-mCherry (200 nL) were microinjected into the PeFLH (anterior–posterior, AP: −1.7 mm; mediolateral, ML: +0.85 mm; and dorsoventral, DV: −5.0 mm) [17] in Hcrt-Cre mice in anterograde tracing model [43]. AAV2/9-EF1α-DIO-GCaMP6s (200 nL) was injected into the PeFLH and the fiber was implanted in the POA (AP: +0.01 mm, ML: +0.75 mm, and DV: −4.0 mm) in Hcrt-Cre mice in fiber photometry model [17]. The fibers were then fixed to the skull using dental cement.

In the optogenetic model, AAV2/9-EF1α-DIO-ChR2-mCherry, AAV2/9-EF1α-DIO-NPHR-mCherry, or AAV2/9-EF1α-DIO-mCherry (200 nL, each) was microinjected into the PeFLH, and the fiber was implanted in the POA in Hcrt-Cre mice and fixed to the skull with 3 stainless-steel screws, which were used for ECoG recording. The positions of the 3 screws were AP: +2.0 mm, ML: +1.5 mm; AP: +2.0 mm, ML: −1.5 mm (recording electrodes); and AP: −5.5 mm, ML: +1.5 mm (reference electrode) [27].

#### Cannula implantation for neuropharmacology

In the pharmacology mouse model, a dual intracerebral injection cannula with an outer diameter of 0.45 mm, an inner diameter of 0.37 mm, and a center-to-center spacing of 1 mm (RWD Life Science, Shenzhen, China) was implanted bilaterally into the POA and fixed to the skull using 3 stainless-steel screws, which were used for ECoG recording.

#### Opto-tetrode implantation for in vivo electrophysiology

In the in vivo electrophysiology mouse model, AAV2/9-EF1α-DIO-ChR2-EGFP (200 nL) was unilaterally injected into the PeFLH, while AAV2/9-Vgat1-NPHR-mCherry (200 nL) or AAV2/9-PV-Cre combined AAV2/9-EF1α-DIO-NPHR-mCherry (1:1, total 200 nL) was injected into the ipsilateral POA in Hcrt-Cre mice. Subsequently, a 16-channel opto-tetrode was implanted in the POA and then fixed to the skull by dental cement.

The mouse models were allowed to recover 7 to 21 d after surgery. Data from the mouse model with untargeted placement of fiber or lacking virus expression were excluded.

### Performance of immunohistochemistry

Mice were deeply anesthetized with 2% isoflurane for at least 5 min and then cardially perfused with saline and 4% paraformaldehyde (PFA). Brains were dehydrated with 30% sucrose in phosphate-buffered saline (PBS) serially following post-fixation in 4% PFA at 4 °C. Brain regions containing the POA and PeFLH were coronally sectioned in 40-μm slices by using a cryostat microtome (Leica, CM1900, Germany). The in vitro brain slices were directly used for immunofluorescence staining after whole-cell patch recording. PBS was used to wash the brain slices 3 times (10 min each). Then, brain slices were blocked by 5% normal donkey serum (NDS) and 0.3% Triton X-100 (PBST) in PBS for 2 h at room temperature. Primary antibodies in PBST and 2.5% NDS were incubated at 4 °C for 24 h, and incubated secondary antibodies in 2.5% NDS in PBST at room temperature for 2 h. Subsequently, brain slices were washed 3 times (10 min each) in PBS. Brain slices were mounted and at last they were imaged by a laser confocal fluorescence microscope (FV1200; Olympus, Japan).

Primary antibodies include rabbit anti-prepro-orexin antibody (1:500, AB3096; EMD Millipore Corporation, USA) and rabbit anti-γ-aminobutyric acid antibody (1:400, GTX125988; GeneTex, USA). Secondary antibodies include Alexa Fluor 488 anti-Rabbit (1:800, 144917; Jackson, USA).

Following background subtraction, images were binarized using a pixel-intensity threshold that was held constant across all samples. Regions of interest (ROIs), defined by background signal, were then manually delineated. Axon density was subsequently quantified as the percentage of red pixels within each ROI relative to its total pixel count, i.e., ((red pixels in ROI) / (total pixels in ROI)) × 100. The POA was anatomically defined as extending from 0.50 mm anterior to 0.23 mm posterior to bregma. Coronal POA sections located near bregma were used to quantify the regional distribution of orexinergic axons.

### Fluorescence in situ hybridization

To determine the general and cell-type-specific expression of orexin receptors in the POA, fluorescence in situ hybridization was performed to detect OX1R and OX2R transcripts together with markers of GABAergic, glutamatergic, and parvalbumin-positive neurons. A total of 32 brain slices were collected from 4 male C57BL/6J mice aged 8 weeks. Brains were dehydrated sequentially with 10%, 20%, and 30% sucrose in PBS serially following 24-h post-fixation in 4% PFA at 4 °C. Brain regions containing the POA were coronally sectioned in 10-μm slices by using a cryostat microtome (Leica, CM1900, Germany). The probes targeting OX1R (Hcrtr1, Cat number: 2307771-B1), OX2R (Hcrtr2, Cat number: 3872851-B5), GABAergic neurons (Slc32al, Cat number: 223481-B2), glutamatergic neurons (Slc17a6, Cat number: 1409191-B2), and PV neurons (Pvalb, Cat number: 192931-B2) were obtained from Pinnoease Corporation (Guangzhou, China). Hybridization and signal amplification were performed using the PinpoRNA RNA Fluorescent Multiplex Assay according to the manufacturer’s protocol. Cell nuclei were counterstained with 4′,6-diamidino-2-phenylindole stain. Images were acquired using a laser confocal fluorescence microscope (FV1200; Olympus) under identical acquisition settings. Co-localization was quantified as OX1R or OX2R positive and neuronal marker positive in 8 POA sections from 4 mice per group. Image acquisition and/or quantification was performed under blinded conditions.

### Isoflurane anesthesia and arousal protocol

A horizontal cylinder (30 cm in length and 15 cm in diameter) was used for mice habituation at least 30 min. Following 600 s wake baseline recording, 1.4% isoflurane in mixed oxygen–air (air/oxygen 2:1) at a flow rate of 1 L/min was used for anesthesia induction and maintenance. After 1,800 s isoflurane perfusion, 1,200 s emergence state was recorded. When the mouse could no longer turn itself prone onto all 4 limbs by turning the cylinder 90° every 15 s, the LORR was defined. The time from the onset of anesthesia to LORR was recorded as the induction time. When the mouse could independently turn from the supine position, with at least 3 paws reaching the bottom of the cylinder by turning the cylinder 90° every 15 s, the RORR was defined. The interval from the termination of isoflurane anesthesia inhalation to RORR was recorded as the emergence time [27].

A heating blanket set at 37 °C was used to keep the body temperature of mice stable around the cage during all in vivo anesthesia experiments.

### Fiber photometry recording and data analysis

To track changes in orexinergic afferent activity during isoflurane anesthesia and arousal process, fiber photometry was used. A fluorescence photometer (Thinker Tech, Nanjing, China) was used to attach the optical fiber. An intensity of 30 to 40 μW 488-nm LED light beam was used to evoke and record GCaMP6s signals of orexinergic terminal during isoflurane anesthesia and arousal. The protocol contains 600 s wake baseline, 1,800 s 1.4% isoflurane anesthesia, and 1,200 s emergence following cessation of isoflurane anesthesia. Meanwhile, the time of LORR and RORR of each mouse were recorded. The data were analyzed by MATLAB R2024b (MathWorks, Natick, MA). The Δ*F*/*F* was calculated as (*F* − *F*_0_)/*F*_0_, where *F*_0_ and *F* are the baseline and the real-time fluorescence signal to normalize the fiber photometry data. The mean GCaMP6s signal (Δ*F*/*F*) was calculated over 30-s windows immediately preceding and following the LORR and RORR events [27].

Half-time: Normalized GCaMP6s signal trace of each mouse was analyzed using GraphPad Prism with fitting Boltzmann sigmoidal curve using the following equation:

$$y=\text{minimum}+\frac{\text{maximum}-\text{minimum}}{1+\frac{t{½}^{-x}}{\text{slope}}}$$ (1)

In the equation, *y* is the mean GCaMP6s signal and *x* is time. The half-times of the curves (*t*½) were used for the comparison of GCaMP6s signal change lags during LORR and RORR processes.

### Optogenetic stimulation protocols

To study the effects of modulation of orexinergic afferents in the POA during isoflurane anesthesia and arousal process, optogenetics was used. Laser intensity at the fiber tip was measured and maintained at 10 to 15 mW. A 473-nm laser at 20 Hz for 20 ms and a 594-nm laser at 1 Hz for 1 s (Thinker Tech) were used for optogenetic activation and inhibition, respectively. Optogenetic stimulation was performed with a cycle of 1 s on and 1 s off to avoid tissue damage while observing induction and emergence times. Optogenetic stimulations were performed for 60 s at 20 min following the start of isoflurane perfusion in observing BSR changes in ECoG. During in vivo electrophysiology experiments, optogenetic stimulations were performed for 30 s at 20 min after the start of isoflurane perfusion.

### Recording and analysis of ECoG

The PowerLab 16/35 amplifier system (PL3516) and LabChart Pro version 8.1.13 (MLU60/8; AD Instruments) were used to record ECoG signals. The sampling frequency of raw ECoG data were set at 1,000 Hz and 0.3 to 50 Hz bandpass-filtered for further analysis. For BSR calculation, the ECoG signals were additionally bandpass filtered at 5 to 30 Hz using a fourth-order Butterworth filter. Suppression periods, which were clearly distinguishable in the ECoG recordings, were manually identified by an experimenter blinded to the viral expression in each mouse. For each mouse, individual upper and lower amplitude thresholds were determined from the maximum and minimum amplitudes of the manually labeled suppression periods. The resulting thresholds in the optogenetic experiments ranged from ±0.014 to ±0.026 mV. The filtered ECoG signal was then transformed into a binary series of burst and suppression states. Periods in which the ECoG amplitude remained within the individually determined suppression thresholds for at least 0.5 s were classified as suppression events and assigned a value of 1, whereas the remaining periods were classified as burst events and assigned a value of 0. The BSR was calculated as the percentage of suppression time within the total recording period using MATLAB R2023b (MathWorks, Natick, MA, USA).

### Neuropharmacology and drug administration

Pharmacological interventions were used to investigate the receptor mechanisms. A concentration of 333 pmol/μL of orexin-A and orexin-B (Tocris Bioscience, Ellisville, USA) was dissolved in saline. Also, SB334867 and TCS-OX2-29 (Tocris Bioscience) were dissolved to 33.3 μg/μL in 5% DMSO [18,27]. The speed of drug microinjection was set at 0.06 μL/min with a total volume of 0.3 μL. Drugs were injected into the POA 15 min before isoflurane inhalation to observe the LORR of mice. Drugs were administered 15 min before the cease of isoflurane inhalation to observe the RORR. Drugs were injected into the POA 15 min following isoflurane inhalation for analysis of BSR.

### In vitro electrophysiology

Briefly, mice were anesthetized by 1.4% isoflurane and decapitated. The brain of mice was immediately dissected. Brains were then transferred to an ice-cold cutting solution containing (in mmol/L) 92 *N*-methyl-D-glucamine, 2.5 KCl, 1.25 NaH_2_PO_4_, 30 NaHCO_3_, 20 HEPES, 25 glucose, 2 thiourea, 5 Na-ascorbate, 3 Na-pyruvate, 0.5 CaCl_2_, and 10 MgSO_4_ (oxygenated with 95% O_2_ and 5% CO_2_) and were then sliced coronally at a thickness of 300 to 350 μm using a vibratome (VT1200S; Leica). According to the anteroposterior extent of the POA, 1 to 2 POA-containing slices were obtained from each mouse, and 3 to 5 neurons were recorded from each slice, corresponding to approximately 3 to 10 neurons per mouse. POA-containing slices were recovered in a submerged chamber and continuously superfused with oxygenated artificial cerebrospinal fluid (aCSF) at 34 °C for 40 to 50 min. The slices were then kept at room temperature until recordings [27,44,45]. The aCSF consisted of (in mmol/L) 124 NaCl, 2.5 KCl, 1.25 NaH_2_PO_4_, 24 NaHCO_3_, 12.5 glucose, 5 HEPES, 2 CaCl_2_, and 2 MgSO_4_ (pH 7.4, osmolality of 300 to 305 mOsm). During recordings, slices were fixed in a recording chamber continuously perfused with oxygenated aCSF at a rate of 3 to 5 mL/min. Micropipettes (4 to 6 MΩ) were prepared from borosilicate glass capillaries (1.5 mm outer diameter and 0.86 mm inner diameter) with a horizontal puller (P-97; Sutter Instruments). Micropipettes were filled with a pipette solution containing potassium ions for whole-cell recording (in mmol/L): 145 K-Gluconate, 10 HEPES, 1 EGTA, 2 Mg-ATP, 0.3 Na_2_-GTP, and 2 MgCl_2_ at pH 7.3, and osmolality of 280 to 290 mOsm. Cells were stabilized for 5 min after whole-cell recording was performed, and AP firing was recorded; for neurons without spontaneous firing, a positive current (5 to 30 pA) was injected to induce steady firing activity. OA (1 μM) or OB (1 μM) was bath applied for 10 min after a 5-min baseline recording. The frequency of AP recorded from the 5-min baseline and the last 5 min of drug application and the last 5 min of washing for comparison was averaged. Data were low-pass filtered at 2 kHz and then digitized at 10 kHz. Online acquisition was performed using pCLAMP 10.6 (Clampex) software (Axon Instruments).

### In vivo electrophysiology

In vivo electrophysiological signals were recorded by using the Blackrock system (Kesheng Ltd., Beijing, China). To capture neuronal spikes and slower LFP and ECoG signals, a bandpass filter (250 to 5,000 Hz) and a low-pass filter (250 Hz) were used, respectively. Offline Sorter software (KlustaKwik, USA) was used to sort single-unit spikes according to a threshold and shape detector using principal component analysis. To identify GABAergic and PV neuronal firings in the POA, a 594-nm laser (1 Hz) was applied during the wake state for 10 s. During PV-neuron-related in vivo experiments, short-duration APs, relatively high rapid-firing, non-bursting activity, were used to distinguish POA GABAergic spikes [46,47]. The clusters which showed a significant reduction of firing rate (>90%) during laser inhibition were identified as POA GABAergic neurons. To analyze neuronal firings, ECoG, and POA LFP, NeuroExplorer (version 5.0) was used. The frequency bands of ECoG and LFP were classified as delta (0.3 to 4 Hz), theta (4 to 10 Hz), alpha (10 to 15 Hz), beta (15 to 25 Hz), and gamma (25 to 50 Hz).

To examine the relationship between POA PV-positive neuronal firings and cortical oscillatory states, coherence was calculated between the spike trains of optogenetically identified PV-positive neurons and simultaneously recorded frontal cortical ECoG signals during wakefulness, stable isoflurane anesthesia, and optogenetic activation of orexinergic terminals in the POA. Coherence was quantified within the delta (0.3 to 4 Hz), theta (4 to 10 Hz), alpha (10 to 15 Hz), beta (15 to 25 Hz), and gamma (25 to 50 Hz) frequency bands using coherence analysis method in NeuroExplorer (version 5.0). A total of 28 PV-positive neurons recorded from 8 mice were included in this analysis.

Half-time: Normalized mean firing rate trace of each neuronal firing was analyzed in GraphPad Prism with Boltzmann sigmoidal curve fitting using the following equation:

$$y=\text{minimum}+\frac{\text{maximum}-\text{minimum}}{1+\frac{t{½}^{-x}}{\text{slope}}}$$ (2)

where *y* is the neuronal firing rate and *x* is the time point. The half-times of the curves (*t*½) were used for comparison of firing rate change lags between POA PV^+^ and PV^−^ neuronal firings during laser inhibition and orexinergic activation processes.

### Sample size determination

Sample sizes were determined with reference to previous studies [27] and computed through a Calculator from https://powerandsamplesize.com/Calculators/. In detail, a sample size of 4 subjects per group would have 80% power to detect an ECoG difference with a significance level of 0.05 using a 2-tailed analysis, so more than 6 mice each group were used in current study. Calculating sample sizes with a significance level of 0.05 and 80% power by using a 2-tailed analysis, more than 5 mice each group were used in anesthesia behavioral experiments, more than 4 mice each group were used for fiber photometry experiments, more than 4 mice each group were used for in vivo electrophysiology experiments, and more than 3 mice each group were used for histomorphology. Also, for in vitro electrophysiology, more than 4 mice each group were used in each experiment.

### Statistical analysis

For statistical analysis, we used GraphPad Prism 8.0.1 (CA, USA). Shapiro–Wilk tests were used to confirm the normality of all data before further statistical analysis. In vitro and in vivo neuronal discharge data and fiber photometry signal data were expressed as the mean and standard error of the mean [SEM] and were analyzed with Student *t* test, 2-way analysis of variance (ANOVA), or 1-way ANOVA. Behavioral data were expressed as the mean and standard deviation [SD] and were analyzed with Student *t* test or ANOVA. Power of ECoG and LFP were expressed as the mean and SEM and were analyzed with 2-way ANOVA. Statistical significance was set at *P* <0.05 in the current study. Sex-related differences in POA GABAergic neuronal firing across consciousness states were analyzed using 2-way ANOVA.

## Data Availability

The authors confirm that the data supporting the findings of this study are available within the article.
