# Supplementary material for: Feedforward Inhibition Mediated by Preoptic Area Parvalbumin Neurons Contributes to Orexin-Induced Active Arousal from Anesthesia
Source: Research (Wash D C). 2026 Nov 5;9:1474. doi: 10.34133/research.1474 (PMC13735207; doi:10.34133/research.1474)
Supplement: Supplementary file 1 [file research.1474.f1.docx]

**Supplementary Table 1. Usage of in the experiments**

| Experiments | Mouse line | Male (valid data) | Female (valid data) | Male (data excluded) | Female (data excluded) |
| --- | --- | --- | --- | --- | --- |
| Anterograde tracing | Hcrt-Cre | 0 | 3 | 0 | 0 |
| Orexin receptors FISH | C57BL/6J | 2 | 2 | 0 | 0 |
| Fiber photometry | Hcrt-Cre | 4 | 2 | 0 | 1 |
| Optogenetic modulations | Hcrt-Cre | 24 | 24 | 2 | 3 |
| Neuropharmacology | C57BL/6J | 45 | 45 | 2 | 1 |
| Multiplex FISH | C57BL/6J | 6 | 6 | 0 | 0 |
| *In vitro* electrophysiology | C57BL/6J | 3 | 4 | 1 | 1 |
| *In vivo* electrophysiology | Hcrt-Cre | 8 | 9 | 1 | 3 |
| Total |  | 92 | 95 | 6 | 9 |
| Overall Total |  | 187 | | 15 | |

**Supplementary Figure 1. Identification of neuronal types in in vitro patch-clamp recordings**

(a) Schematic of the whole-cell patch-clamp recording configuration in coronal brain slices containing the POA. (b) A representative recorded neuron was filled with biocytin (red) and subsequently confirmed to be GABA-positive (green) by immunofluorescence staining.

**Supplementary Figure 2. Sex-specific alterations in firing rates of POA GABAergic neurons during isoflurane anesthesia and arousal**

(a) Sex-specific raster plot of POA GABAergic neuronal firings across three different states. (b) Quantification the firing rate difference of sex-specific POA GABAergic neurons across the three states. Statistical analysis: Two-way ANOVA followed by Bonferroni's multiple comparisons test was used in this figure.

**Supplementary Figure 3. EEG and POA LFP changes during isoflurane anesthesia process and during optogenetic activation of orexinergic terminals**

(a) Left: Power spectral density of POA LFP during Wake, ISO, and Emergence. Right: Quantification of relative power in distinct frequency bands, showing an increase in delta (δ) power and a decrease in theta (θ) power under anesthesia. (b) Left: Power spectral density of cortical EEG. Right: Quantification of relative power in distinct frequency bands, mirroring the changes observed in the POA LFP. (c) Left: Power spectral density of POA LFP before (Pre) and during (Laser) optogenetic stimulation. Right: Quantification of relative power changes across different frequency bands, showing an increase in delta (δ) and decreases in alpha (α), beta (β), and gamma (γ) power. (d) Left: Power spectral density of EEG before (Pre) and during (Laser) optogenetic stimulation. Right: Quantification of relative power changes across different frequency bands, showing an increase in delta (δ) and decreases in alpha (α), beta (β), and gamma (γ) power. * *P* < 0.05, ** *P* < 0.01, *** *P* < 0.001. Statistical analysis: Two-way ANOVA followed by Bonferroni's multiple comparisons test was used in this figure.

**Supplementary Figure 4. Changes of coherence of PV^+^/PV^-^ neuronal spikes and ECoG during isoflurane anesthesia and arousal process**

(a) Quantification of coherence of POA PV^+^/PV^-^ neuronal spikes and ECoG during three different states. (b) Traces (left) and quantification (right) of coherence of POA PV^-^ neuronal firing and frontal cortex ECoG during three different states. * *P* < 0.05, ** *P* < 0.01. Statistical analysis: Two-way ANOVA followed by Bonferroni's multiple comparisons test was used in this figure.
